# Assessment of Eye Care Apps for Children and Adolescents Based on the Mobile App Rating Scale: Content Analysis and Quality Assessment

**DOI:** 10.2196/53805

**Published:** 2024-09-13

**Authors:** Ming Liu, Xiaoqian Wu, Ziyu Li, Dongmei Tan, Cheng Huang

**Affiliations:** 1 College of Medical Informatics Chongqing Medical University Chongqing China; 2 Department of Quality Management Daping Hospital Army Medical University (The Third Military Medical University) Chongqing China

**Keywords:** Mobile apps, Eye care, Myopia, MARS, Children, Adolescent, Quality Assessment, Content Analysis, mhealth, China

## Abstract

**Background:**

In China, the current situation of myopia among children and adolescents is very serious. Prevention and control of myopia are inhibited by the lack of medical resources and the low awareness about eye care. Nevertheless, mobile apps provide an effective means to solve these problems. Since the health app market in China is still immature, it has become particularly important to conduct a study to assess the quality of eye-care apps to facilitate the development of better eye-care service strategies.

**Objective:**

This study aimed to evaluate the quality, functionality, medical evidence, and professional background of eye-care apps targeting children and adolescents in the Chinese app stores.

**Methods:**

A systematic search on iOS and Android app stores was performed to identify eye-care apps for children and adolescents. The general characteristics, development context, and functional features of the apps were described. Quality assessment of the apps was completed by 2 independent researchers using the Mobile App Rating Scale.

**Results:**

This study included 29 apps, of which 17 (59%) were developed by commercial organizations and 12 (41%) had a design with relevant scientific basis. The main built-in functions of these apps include self-testing (18/29, 62%), eye exercises (16/29, 55%), and eye-care education (16/29, 55%). The mean overall quality of eye-care apps was 3.49 (SD 0.33), with a score ranging from 2.89 to 4.39. The overall Mobile App Rating Scale score exhibited a significant positive correlation with the subscale scores (*r*=0.81-0.91; *P*<.001). In addition, although most apps provided basic eye-care features, there are some deficiencies. For example, only a few apps were developed with the participation of medical organizations or professional ophthalmologists, and most of the apps were updated infrequently, failing to provide the latest eye-care information and technology in a timely manner.

**Conclusions:**

In general, the quality of eye-care apps for children and teenagers in Chinese app stores is good. These apps fulfill users’ needs for eye-care services to a certain extent, but they still suffer from insufficient medical background, low user engagement, and untimely updates. In order to further improve the effectiveness of eye-care apps, cooperation with medical institutions and professional ophthalmologists should be strengthened to enhance the scientific and authoritative nature of the apps. At the same time, interactive features and regular updates should be added to enhance user participation and the continuity of the apps. This study provides a reference for future development or improvement of eye-care apps, which can help promote myopia prevention and control.

## Introduction

Children and adolescents are currently facing complex and serious health challenges, such as obesity, myopia, and anxiety and depression [1,2]. Children and adolescents represent the future of society; thus, more attention should be paid to their growth and development. Nearsightedness has been recognized as the most common eye disorder among children and adolescents [3]. Patients with shortsightedness experience blurred vision at a distance. High myopia is also associated with pathologic myopia, which can lead to complications such as retinal tear and detachment, macular degeneration, and irreversible vision damage [4]. Meanwhile, there is a link between shortsightedness, astigmatism, anisometropia, and amblyopia, which may interact with each other and work together to affect the visual health of children and adolescents [5,6].

A government statistical report in 2021 stated that the overall incidence of nearsightedness among Chinese children and adolescents was 52.7% and that the number of patients was about 100 million; shortsightedness is highly prevalent among children and adolescents and shows a trend of decreasing age of incidence [7]. However, prevention and control of myopia are hindered by insufficient medical resources, regional limitations, insufficient existing knowledge of healthy eye use, and low awareness about myopia [8-10]. Nevertheless, mobile health (mHealth) apps bring a new possibility to solve the aforementioned problems [10,11].

With their robust functionality, mobile apps have become a valuable tool for improving health care delivery and providing low-cost interventions [12,13]. In the domains of hypertension [14], nutrition and diet [15], exercise and medication adherence [16,17], chronic disease management [18,19], and so on, the significant effects of the use of apps on clinical, knowledge, behavioral, and psychosocial outcome improvement have been demonstrated [20,21]. Mobile apps have not only been recognized as an effective means to raise awareness of eye health [22,23], but have also efficiently provided myopia screening services and vision monitoring services [24,25].

Further integration of mobile apps with the internet has also significantly improved mHealth app accessibility [26]. Meanwhile, the number and types of apps have rapidly increased. The use of mobile apps is not limited to a specific time and place [27], which can help effectively overcome barriers such as insufficient health care resources and regional restrictions. In particular, eye-care app enables children and adolescents with no access to specialized eye education due to geographic location and economic status to receive high-quality eye-care services. Eye-care apps have attracted increasing attention with the discovery of the many advantages of mobile apps and the growing demand for eye-care services [28].

Nowadays, studies on the content and quality of eye-care apps are scarce [29]. In a study of eye-care–related apps in Chinese app stores, the researchers assessed the quality of the app through its general characteristics [30]. However, the professional background and built-in functionality of the app are also significant. The built-in functions of an app are an important reference for people to choose them, and the presence or absence of a professional background affects the user’s trust on the app [31-33]. Due to the immature health app market in China [34], it becomes particularly important to conduct assessment studies on the quality of eye-care apps to facilitate the development of better strategies for eye-care services [35,36].

Therefore, this study aimed to summarize the general characteristics of eye-care apps for children and adolescents in Chinese app stores and to assess the quality of these apps. This will help users identify high-quality eye-care apps and provide a realistic basis for app development and improvement.

## Methods

### Systematic Search Strategy

In this study, a systematic search was performed on Chinese app stores to identify eye-care apps applicable to children and adolescents on February 15, 2023. At present, the sources of app stores are mainly categorized into third party companies and mobile phone manufacturers. According to the comprehensive ranking of Chinese app stores [37], Huawei AppGallery is the largest Android app store, and My app is the largest third-party app store in China. Therefore, Apple App Store (iOS) as well as Tencent My App and Huawei AppGallery (Android) were searched.

Through literature search and group discussion, the following keywords were used: eye care, myopia, eye health, vision, adolescent eye care, eye protection, and childhood myopia. Combining the age definition of child and adolescent as well as the age-applicable interpretation of app stores, we have added review criteria for targeted apps. The aforementioned keywords were searched in the app stores using an account that was not logged into any user, and all search results were recorded. If an app existed on both iOS and Android with identical design and content, considering the dominance of Apple App Store in app development [38], we evaluated the iOS version of the app.

### Eligibility Criteria

After eliminating duplicate apps from the recorded search results, 2 researchers performed 2 rounds of app screening based on software description and usage. In the first round of screening, apps that satisfied all of the following conditions were included (Textbox 1):

**Inclusion criteria**

- The topic and content of the apps are related to eye care and the apps provide eye-care services
- The target audience of the apps is people needing eye-care services
- The target users of the apps are people aged 18 years or younger, and
- The apps are available in Chinese version

**Exclusion criteria**

- The apps focus on content unrelated to eye-care services, such as games, timers, and brightness adjustment on cell phones that do not provide eye care
- The apps are targeted only at eye-care service providers, such as doctors, nurses, research scholars, and other health professionals
- The apps do not have a Chinese version

In the second round of screening, apps were excluded if they were not successfully downloaded (after 2 attempts) or could not be logged in for use due to technical errors or special authentication.

### Information Extraction

Information of the apps was extracted by 2 researchers, which included platform, developer, applicable age, last update time, star rating, number of downloads, and professional development background (Table 1). The general characteristics of the apps were described based on the information of the apps was extracted. Furthermore, through literature review and group discussion, the main functions of the eye-care apps were categorized into self-testing, eye exercises, monitoring of eye use data, eye-care education, patient record management, physical eye protection, medical consultation, online community, and eye-care mall. All disagreements were resolved through discussion between the researchers.

**Table 1:** General information collected for each app.

Assessment measure	Definition and values
Platform	Huawei (AppGallery), Apple (Apple App Store), Tencent (My App).
Developer	Commercial organizations, professional ophthalmology organization, individual developer.
Applicable age	Children (12 years or younger), adolescents (13-17 years).
Last update time	The latest update time when retrieving.
Star rating	Star rating (out of 5) left by users in the app store.
Downloads	Number of app downloads in the app store.
Evidence-based and professional background	Does the app claim that the design is based on relevant eye care theory, academic research, or the opinions of health professionals?

### App Quality Assessment

To assess the quality of the apps more objectively, the Mobile App Rating Scale (MARS), a reliable tool for classifying and assessing the quality of mHealth apps [39], was used. MARS has been used to assess the quality of different apps, such as mental health [27], nutritional and diet-related [40,41], and chronic disease management [42,43] apps. It consists of 4 objective quality subscales of engagement, functionality, aesthetics, and information quality and 1 subjective quality subscale. As this study aimed to objectively assess the quality of apps, the subjective quality subscales were discarded. Before the formal scoring, 2 researchers randomly selected 5 apps to determine the MARS scoring rules so as to ensure that their understanding of the MARS questions and scoring criteria was consistent. To fully experience the services offered by the apps, the 2 researchers downloaded and used each app for at least 20 minutes during the formal scoring.

### Data Analysis

To ensure the quality assessment reliability of the 2 researchers, within-group correlation coefficients (2-way random, average measure and absolute agreement) were used to assess researchers’ agreement at the subscale and overall score level [44]. Pearson’s correlation coefficients were used to explore the correlation among the overall MARS score, subscale scores, and user ratings. All statistical analyses were conducted in SPSS (version 26, IBM Corp).

## Results

### App Selection

**Figure 1:**
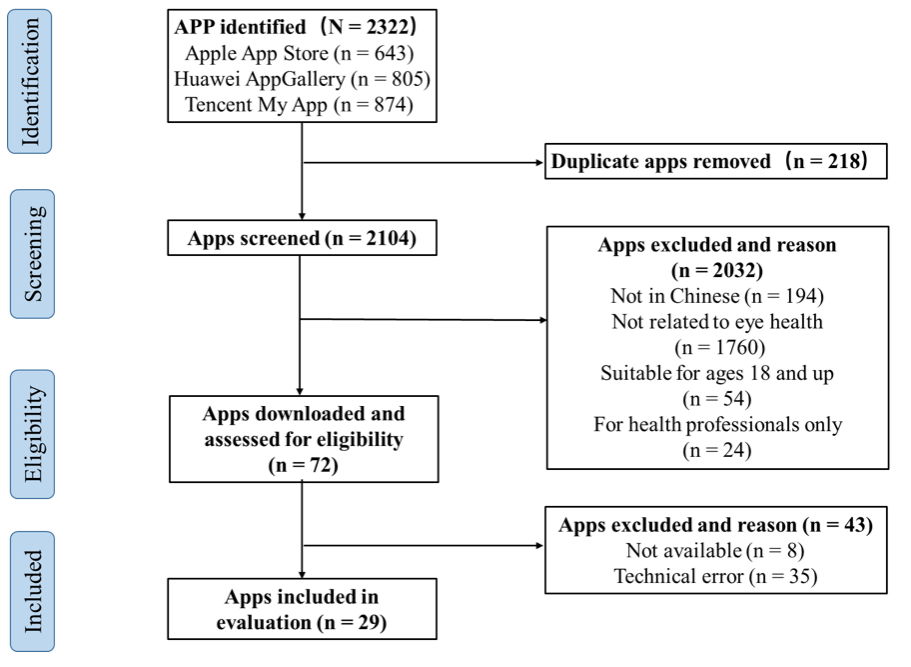
Flowchart for the systematic search and selection of apps.

Through keyword search, 2322 apps were identified. Tencent My Apps had the highest number of apps (n=874, 37.6%), followed by Huawei AppGallery (n=805, 34.7%) and then Apple App Store (n=643, 27.7%). First, the search results of the apps were consolidated, and 218 apps were excluded due to repeated occurrence. Subsequently, 2032 (87.5%) apps were also excluded based on the first round of screening criteria. The second round of operability screening was performed on the remaining 72 apps. Of the apps excluded in this round, 35 (48.7%) could not be downloaded or properly used due to technical reasons and 8 (11.1%) required special authentication to log in for use. Finally, after 2 rounds of screening, 29 (40.2%) apps were included in the study (Figure 1).

### General Characteristics of the App

The sources of the included apps were counted, and it was found that 18 (62%) apps were from Apple App Store, 8 (28%) from Tencent My Apps, and 3 (10%) from the Huawei AppGallery. Furthermore, more than half of the apps (17/29, 58%) were developed by commercial organizations, 8 (28%) by individual developers, and 4 (13%) by professional ophthalmology organizations. Simultaneously, based on the update time of the apps, 3 (10%) apps were updated within 1 month; 16 (55%), more than 1 month but less than 1 year; and 10 (34%), before 1 year (Table 2).

**Table 2:** Descriptive information on the apps reviewed.

Assessment measure		Apps, n (%)
**Platform**		
	Apple App Store	18 (62)
	Huawei AppGallery	3 (10)
	Tencent My App	8 (28)
**Developer**		
	Commercial organizations	17 (59)
	Professional ophthalmology organizations	4 (13)
	Individual developer	8 (28)
**Applicable age**		
	Children	23 (80)
	Adolescents	29 (100)
**Last updated**		
	<1 month	3 (10)
	>1 month and <1 year	16 (55)
	>1 year	10 (35)
**Evidence-based and professional background**		
	Participation of professional ophthalmologists	8 (28)
	Verified methods and theories	3 (10)
	Peer-reviewed academic research	1 (3)
	Not mentioned	17 (59)

### Evidence-Based and Professional Background

The details of the apps were checked, which indicated that 17 (59%) of them did not have an app design with a relevant scientific basis. As for the remaining 12 (41%) apps, their details indicated that their design was based on relevant scientific foundation: 8 (28%), designed with the involvement of medical professionals (eg, clinicians, ophthalmologists); 3 (10%), designed using a validated methodology such as ocular muscle training; and 1, designed based on scientific basis coming from peer-reviewed academic research.

### Functional Review

The results of the statistical analysis indicated that eye-care apps provide a wide range of eye-care services for children and adolescents. More than half of the apps offered functions including self-testing (18/29, 62%), eye exercises (16/29, 55%), and eye-care education (16/29, 55%). About a quarter of the apps offered functions including patient record management (10/29, 34%), online community (7/29, 24%), and monitoring of eye use data (7/29, 24%). Furthermore, less than a quarter of the apps offered functions such as eye-care mall (6/29, 21%), medical consultation (6/29, 21%), and physical eye protection (5/29, 17%; Multimedia Appendix 1). The most common combination among multifunctional apps was that of self-testing and eye-care education (12/29, 41%), followed by a combination of self-testing and eye exercises (7/29, 24%).

### Self-Testing

The self-testing function is mainly used to detect the user’s vision level and other eye disorders. The vision testing function (18/18, 100%) was developed based on the E Vision Scale or C Vision Scale. The users participated in the test through manual selection as well as gesture and voice recognition. Meanwhile, the eye disease screening function (10/18, 56%) includes screening for color blindness, color deficiency, astigmatism, macular degeneration, and other diseases. Regular visual acuity screening of children and adolescents provides timely information on the current status of their vision and enables detection of eye disorders such as astigmatism, macular degeneration, and refractive errors.

### Eye Exercises

A total of 16 apps offered the eye exercise function. The services included in this function were eye relaxation exercises, eye focus training, and amblyopia correction training. Eye exercise function is presented in the form of animations (12/16, 75%), games (6/16, 37.5%), and eye exercises (5/16, 31%). As one of the most important functions in eye-care apps, whether or not the eye exercise function has a scientific basis affects users’ trust and willingness to use it. After reading the introduction of the apps offering eye exercises, only 6 apps were found to have functions based on scientific evidence, such as eye muscle training and the theory of eye exercises. Furthermore, details of the “FireEye” app clearly state that its function is developed based on the results of big data analysis and professional optometry academic research.

### Eye-Care Education

More than half of the apps (16/29, 55%) offer eye-care education function. The types of eye-care information provided include eye-care science articles (16/16, 100%), eye-care news (4/16, 25%), and eye-care instructional videos (7/16, 44%). Articles on eye-care science had the highest readership and attention. These articles focused on how to use eyes healthily and were presented in a combination of text and animation. In addition, based on the quality assessment results of the apps, eye-care education in the form of videos was found to be more attractive owing to its vivid sound and picture effects than eye-care education in the form of text.

### Patient Record Management

The patient record management function is mainly used to record the vision conditions of children and adolescents at different periods. The eye diseases that can be recorded by this function include nearsightedness, hyperopia, strabismus, and amblyopia. A total of 10 apps offered the vision record management function. Further analysis of this function revealed that 6 apps not only recorded basic information, such as name, age, and recording time, but also provided patients with treatment reports or training plans based on the results of the vision records.

### Online Community

The online community function embodies the great advantages of eye-care apps: proximity, interactivity, and convenience. The online community function exists in 7 (24%) apps, providing a social platform for eye care. In this community, doctors, patients, and others can communicate with each other through text and images. This function can effectively solve problems on consultation fees, long waiting time, and communication barriers that may exist in the actual medical service area. When users and doctors interact with each other through the platform, the distance between them is significantly shortened. Furthermore, patients suffering from the same disorder can come together to discuss the symptoms and treatment of the disease through this platform [45].

### Mobile App Rating Scale Evaluation

**Figure 2:**
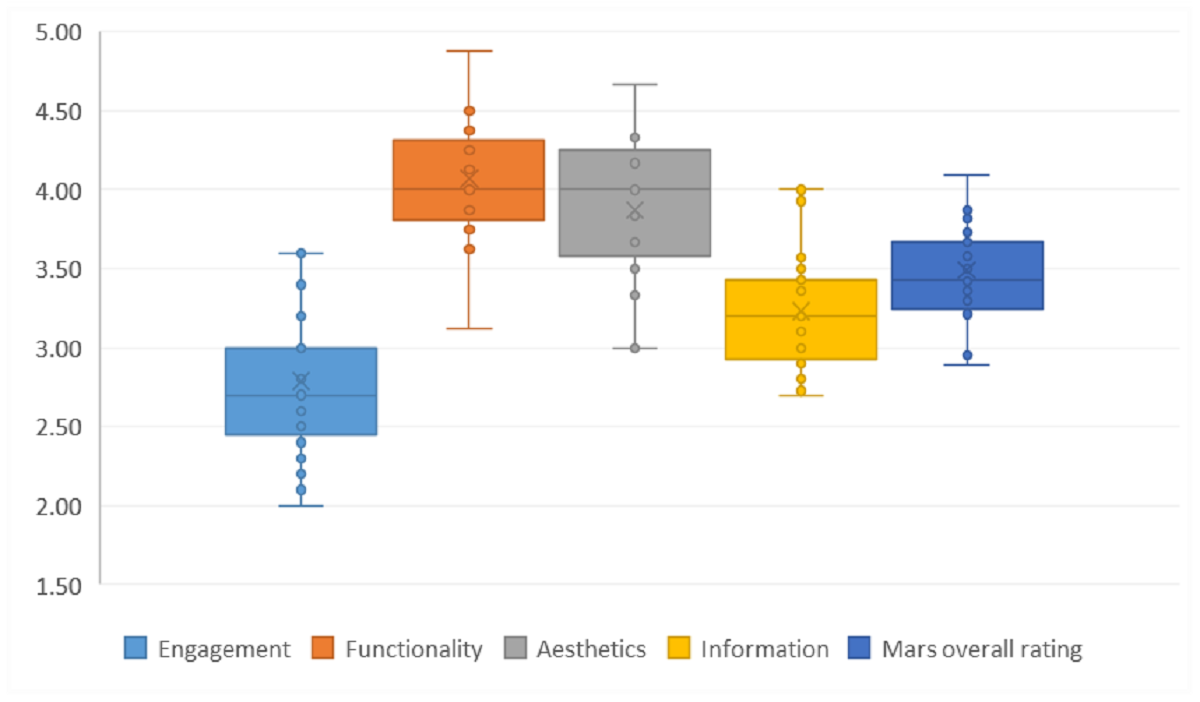
Graphical representation of the distribution of the Mobile App Rating Scale overall and subscale score (N=29).

The overall MARS scores exhibited high inter-reviewer reliability (intraclass correlation coefficient [ICC] 0.95, 95% CI 0.90-0.98). Meanwhile, the 4 subscales demonstrated good consistency: engagement ICC of 0.91 (95% CI 0.82-0.96), functionality ICC of 0.81 (95% CI 0.60-0.91), aesthetics ICC of 0.94 (95% CI 0.87-0.97), and information ICC of 0.90 (95% CI 0.79-0.95). The overall average MARS score of the eye-care apps was 3.49 (SD 0.33). The span of score between the highest scoring app (“Plano Eyes,” 4.39) and the lowest scoring app (“Eye 3D”, 2.89) was 1.5 (on 5-point Likert scale). The average scores for each subscale were as follows: engagement score, 2.79 (SD 0.47); functional score, 4.07 (SD 0.36); aesthetic score 3.87, (SD 0.43); and information score, 3.23 (SD 0.36). The engagement subscale had the largest span of scores (2.0-4.0). In terms of the app score distribution, 2 apps had MARS scores above 4 and 2 apps with scores below 3. Figure 2 presents the distribution of the overall quality of the apps and the scores on the 4D subscales.

### Correlation of Mobile App Rating Scale Scores and User Ratings

Further analysis of the correlation among the MARS overall score, subscale scores, and user ratings on the app store revealed that the MARS overall and subscale scores were positively correlated (*r*=0.81-0.91, *P*<.001). Table 3 summarizes the results of the correlation analysis.

By combining the professional background of the apps and update time, apps with an update time of less than 1 month (3.66) or with a medical professional development background (3.95) were found to have an overall average MARS score higher than the overall average MARS score (3.49). Meanwhile, the overall average MARS score for apps that were updated for more than 1 year (3.33) or did not have a background in medical specialty development (3.39) was lower than the overall average MARS score.

**Table 3:** Correlation between the Mobile App Rating Scale subscale and the overall score and the user star score.

Characteristic	Engagement, correlation	Functionality, correlation	Aesthetics, correlation	Information, correlation	Overall rating. correlation
Engagement	—^a^	—^a^	—^a^	—^a^	—^a^
Functionality	0.40	—^a^	—^a^	—^a^	—^a^
Aesthetics	0.46^b^	0.54^b^	—^a^	—^a^	—^a^
Information	0.73^c^	0.60^c^	0.66^c^	—^a^	—^a^
Overall rating	0.81^c^	0.75^c^	0.82^c^	0.91^c^	—^a^
User star rating^d^	0.33	0.34	0.02	0.33	0.31

^a^Not applicable.

^b^*P*≤.01.

^c^*P*≤.001.

^d^Apps with zero user star ratings were excluded.

## Discussion

### Principal Findings

This study discussed the general characteristics, main functions, and professional context of eye-care apps and also assessed the quality of these apps.

The main functions of the eye-care app included 9 categories: self-testing, eye exercises, monitoring of eye use data, eye-care education, patient record management, physical eye protection, medical consultation, online community, and eye-care mall. In terms of the number of built-in functions in apps, the average number of built-in functions was 3.1. In addition, the app with the most built-in functions contains 6 of the 9 categories of functions. The most common combination of functions was that of vision tests and screenings and eye-care information. The distribution of features in eye-care apps was broadly similar compared with previous studies, with an increase of 0.8 in the average number of built-in features in apps and an increase in the frequency of online community features [30,46,47]. There is a trend toward multifunctionality combination in the development of eye-care apps. Most eye-care apps in Chinese app stores include multiple eye-care–need services in one app. However, in the global app stores, these apps are usually considered to be designed for specific diseases or ophthalmic treatments, such as myopia surgery and cataract surgery [8,48].

In our count of app development backgrounds, only 1 app (FireEye) explicitly stated that its usability had been confirmed by peer-reviewed academic research. However, apps’ lack of medical context can affect users’ trust in them. Summary studies of apps for mental health, chronic disease management, exercise, and medication adherence found that the lack of scientific validation of apps was a recurring issue [14,18,19,22,23]. Of the 29 app developer statistics, 17 (59%) apps were developed by commercial organizations; 4 (13%), by professional ophthalmology organizations; and 8 (28%), by individual developers. These statistics indicated that the lack of involvement of medical professionals or scientific theories in the development of apps is a significant issue. Thus, the involvement of medical professionals in app design and function development is an important means to improve the professionalism of apps [15,40,49].

By counting the last update time of the apps, it was found that only 3 (10%) had been updated within 1 month and 10 (34%) for more than 1 year. Apps that are not updated for a long time may exhibit system vulnerabilities that are more easily exploited by lawbreakers, which in turn threatens the security of users’ personal information [28,50]. By updating apps in a timely manner, developers can not only ensure the security of users’ information but also increase the attractiveness of the apps to users by adding function modules to the apps [27,51].

Through an in-depth exploration of the functions of eye-care apps, one of the apps with a rating of more than 4 (Eye Nurse) has developed intelligence-guided diagnosis function based on artificial intelligence (AI). In this function, users can initially diagnose their disease by selecting age, gender, relevant symptoms, and disease duration. Simultaneously, the AI customer service generates a self-diagnostic report that includes a review of the condition, possible diseases, and doctor’s recommendations. Initial diagnosis of patients using the intelligence-guided diagnosis function can effectively alleviate the problem of insufficient health care resources. Existing research also confirms the feasibility of AI application to the diagnosis and treatment of relevant diseases [52-54]. The overall MARS scores for these apps ranged from 2.89 to 4.39, suggesting that the quality gap for apps was within the normal range [55]. However, these apps had lower mean scores on the engagement and information subscales (2.79, 3.23). This indicates that developers should focus on the improvement of user engagement and information quality during app development. In terms of how to improve user engagement, one of the apps with an engagement score of 4 (Plano Eyes) provided the following strategies: on the one hand, the app designed a punch-card module with daily life tasks, such as outdoor exercise, eye-care habits, and electronic use, which enhances the interaction between the app and the user. On the other hand, the app used audiovisual animations suitable for children and adolescents in eye exercises and eye-care education functions and constructed the eye exercise function in the form of a minigame. Related studies have also demonstrated that the form in which the functions of the apps are presented is an important factor influencing user engagement [56].

The mean MARS score suggests that the overall quality of eye-care apps for children and adolescents in Chinese app stores is acceptable. Meanwhile, by grouping and comparing the 2 factors of update time and presence or absence of a professional background, these 2 factors were found to have a potential association with the quality of the app. Furthermore, there may not be a correlation between user star ratings and MARS, which is consistent with the findings of quality correlation studies with mental health, family assessment, and breast cancer management apps [27,57,58], but food allergy app studies have found a strong correlation [8]. Perhaps the star ratings in the app store came from earlier versions of the app or may have been influenced by other factors and therefore do not fully represent the quality of the app [59]. Previous studies have also found that app quality depends not only on functionality but also on the content and design approach [60].

### Contribution

In this study, a content analysis of eye-care apps for children and adolescents in Chinese app stores was conducted and the quality of these apps was assessed using a scale. Through the results of the study, deficiencies in eye-care apps at this stage, such as insufficient professional medical background, untimely updates, and low user engagement, were identified. At the same time, reference suggestions for the upgrading of eye-care apps were provided. In addition, we found that incorporation of AI technology may lead to technological breakthroughs in preliminary diagnosis.

### Limitations and Future Work

This study has some limitations. We cannot guarantee that all apps are included or that all services (eg, connecting with a certified professional) are provided as requested. Furthermore, the app stores are constantly changing, and apps are on and off the shelves of the app store at all times. In addition, our research was conducted by only 2 researchers; thus, we were unable to advise on app development from the perspective of the target users. Therefore, as a next step, we intend to approach the subjective perspective of users requiring eye-care services to obtain the most realistic views of actual users of eye-care apps. Certainly, evaluation of the efficacy of a certain function in the app, such as relieving eyestrain and self-testing, would also be a good research direction.

### Conclusions

This study analyzed the content and quality of 29 apps in Chinese app stores that are suitable for eye care for children and adolescents. The results of the study indicate that the quality of these apps is acceptable. However, the lack of medical background in apps affects user trust, leads to low app engagement, and reduces user dependence. Furthermore, apps that are not updated for a long time can threaten the security of users’ information. Meanwhile, combining eye-care apps with AI technology may bring about dramatic changes in the popularity and quality of eye-care services in the foreseeable future. These findings provide a realistic basis for the improvement and development of eye-care apps in the future.

## Appendix

Multimedia Appendix 1 Functional review results of the eye care apps.

## Abbreviations

AI: artificial intelligence
ICC: intraclass correlation coefficient
MARS: Mobile Application Rating Scale
mHealth: mobile health
