# Supplementary figures and images for: Assessment of Eye Care Apps for Children and Adolescents Based on the Mobile App Rating Scale: Content Analysis and Quality Assessment
Source: JMIR Mhealth Uhealth. 2024 Sep 13;12:e53805. doi: 10.2196/53805 (PMC11437221; doi:10.2196/53805)

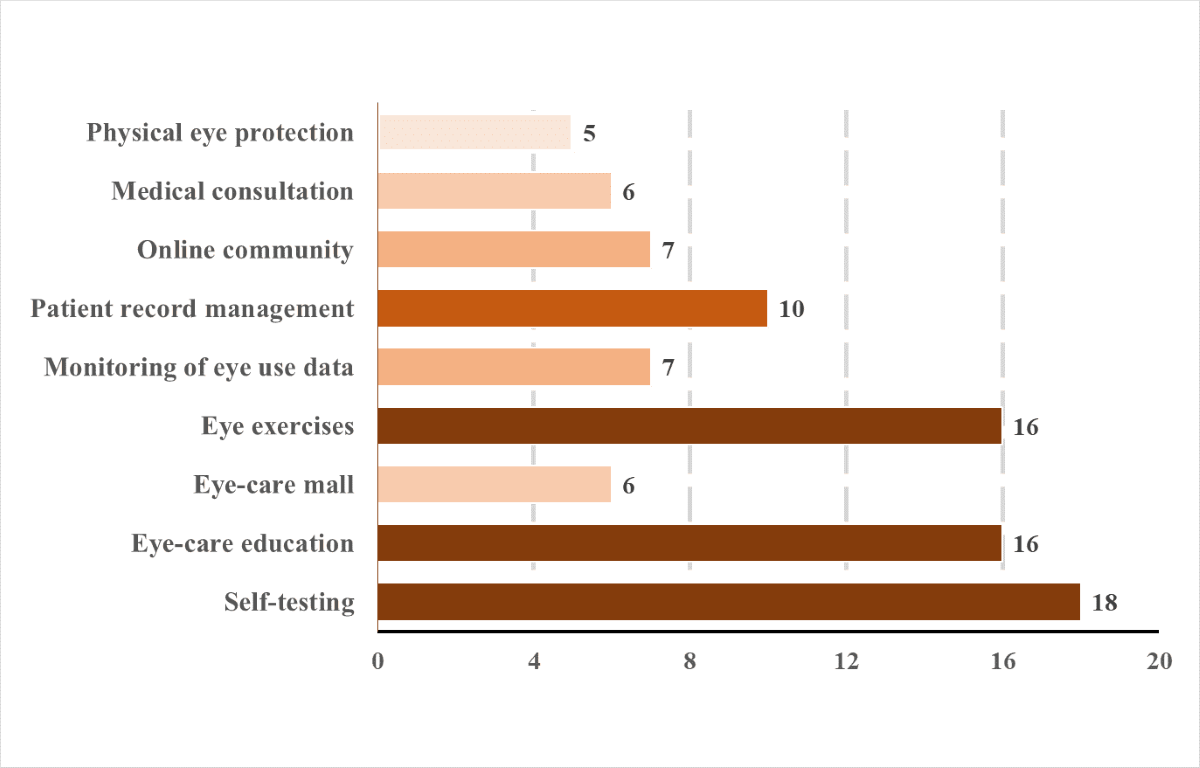

Supplement: Multimedia Appendix 1 [file mhealth_v12i1e53805_app1.png]
